# Plasticity of neutrophils reveals modulatory capacity

**DOI:** 10.1590/1414-431X20154524

**Published:** 2015-06-23

**Authors:** S.M. Perobelli, R.G. Galvani, T. Gonçalves-Silva, C.R. Xavier, A. Nóbrega, A. Bonomo

**Affiliations:** 1 Departamento de Imunologia, Instituto de Microbiologia Paulo de Góes, Universidade Federal do Rio de Janeiro, Rio de Janeiro, RJ, Brasil; 2 Laboratório de Pesquisa sobre o Timo, Instituto Oswaldo Cruz, FIOCRUZ, Rio de Janeiro, RJ, Brasil; 3 Departamento de Ciência da Computação, Universidade Federal de São João Del Rei, São João Del Rei, MG, Brasil; 4 Programa Fiocâncer, Fundação Oswaldo Cruz, Rio de Janeiro, RJ, Brasil

**Keywords:** Autoimmunity, Cancer, Infection, Inflammation, Neutrophil, Suppression

## Abstract

Neutrophils are widely known as proinflammatory cells associated with tissue damage and for their early arrival at sites of infection, where they exert their phagocytic activity, release their granule contents, and subsequently die. However, this view has been challenged by emerging evidence that neutrophils have other activities and are not so short-lived. Following activation, neutrophil effector functions include production and release of granule contents, reactive oxygen species (ROS), and neutrophil extracellular traps (NETs). Neutrophils have also been shown to produce a wide range of cytokines that have pro- or anti-inflammatory activity, adding a modulatory role for this cell, previously known as a suicide effector. The presence of cytokines almost always implies intercellular modulation, potentially unmasking interactions of neutrophils with other immune cells. In fact, neutrophils have been found to help B cells and to modulate dendritic cell (DC), macrophage, and T-cell activities. In this review, we describe some ways in which neutrophils influence the inflammatory environment in infection, cancer, and autoimmunity, regulating both innate and adaptive immune responses. These cells can switch phenotypes and exert functions beyond cytotoxicity against invading pathogens, extending the view of neutrophils beyond suicide effectors to include functions as regulatory and suppressor cells.

## Neutrophil functions: protection, immune regulation, and damage

Known as pro-inflammatory cells, neutrophils are largely associated with tissue damage and are almost always stigmatized as the “bad guys” of the immune system (1). These cells are well known for their arrival at sites of infection, where they exert their phagocytic activity, release their granule contents (e.g., proteases), and destroy the surrounding tissue, Neutrophils die subsequent to performing these functions (2). Recently, evidence has emerged of a long-lived neutrophil subtype with a half-life of 5.4 days, a much longer span than the “traditional” half-life of 7 hours (3,4). A longer half-life allows these cells to switch phenotypes and to exert regulatory functions, thus modifying the view that neutrophils only function as suicide effectors (2).

**Figure 1:**
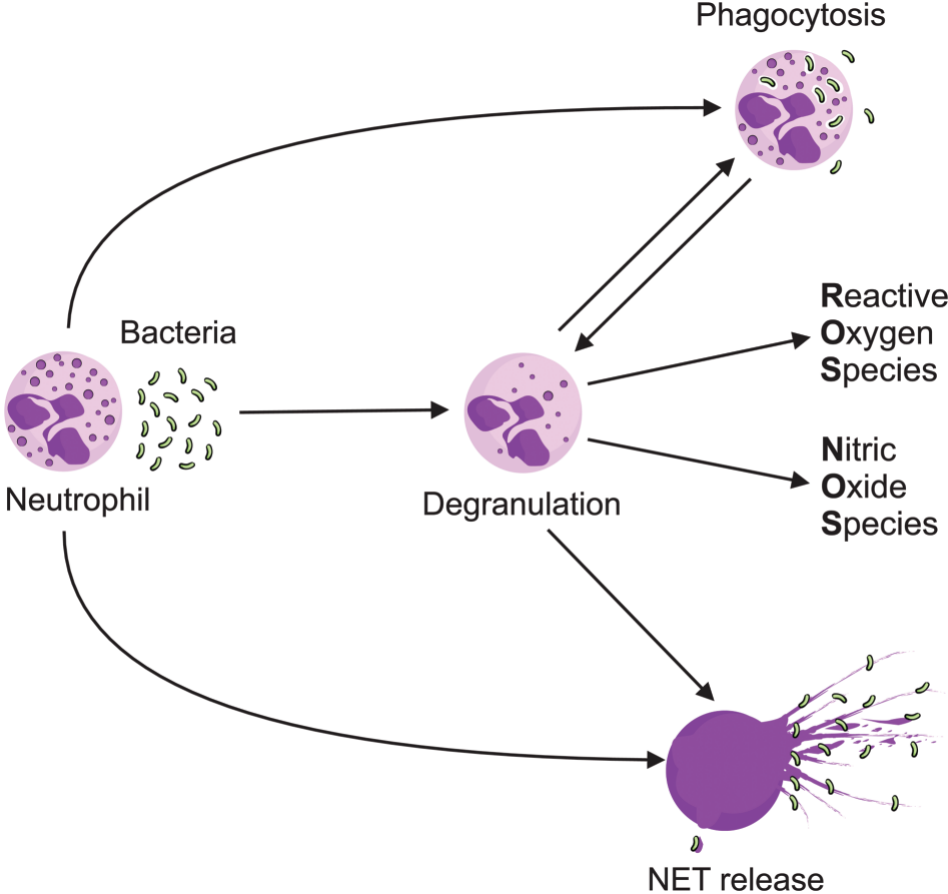
Mechanisms used by neutrophils to control infection. Phagocytosis is a primary mechanism of pathogen elimination. Upon activation, usually through recognition by toll-like receptors (TLRs), neutrophils can degranulate, releasing their granule content. Neutrophils can also release nuclear contents along with the granule contents, “trapping” and killing the microorganism through neutrophil extracellular traps (NETs).

The immune functions of neutrophils involve three key activities. These are production and release of granules that store molecules with active microbicidal activity; generation and release of oxidative bursts, i.e., reactive oxygen species (ROS) such as O_2_^-^, H_2_O_2_, HOCl, and OH; and release of neutrophil extracellular traps (NETs, Figure 1) (5). Defects in either of the first two activities result in severe immunodeficiency such as neutrophil-specific granule deficiencies or chronic granulomatous disease. Moreover, neutropenia can present as a wide range of diseases, from transient suppression to serious systemic diseases. The clinical significance of neutropenia ranges from a mild laboratory abnormality to severe disorders characterized by recurrent life-threatening infections. The nature of the conditions underlines their essential role in innate immunity and resistance to infections (6).

Neutrophil granule contents have potent antimicrobial activity and are also highly cytotoxic (5). There are three types of granules, all of which contain lysozyme and are classified according to their contents: *i*) primary or azurophilic granules, which contain potent hydrolytic enzymes, like elastase and myeloperoxidases (MPOs); *ii*) secondary or specific granules, with high levels of lactoferrin; and *iii*) tertiary or gelatinase granules, which are rich in matrix metalloproteinases (MMPs). Recently, granules with ficolin-1 were described in human neutrophils. Ficolin-1 is primarily located in gelatinase granules but also occurs in gelatinase-poor granules with high exocytic activity. Rapid release of ficolin-1 is followed by binding to microbial surfaces and recognition by molecules in the lectin complement pathway (7,8).

NETs are composed of decondensed chromatin and some materials released from primary, secondary, and tertiary granules, and are expelled from the neutrophils (9). These structures can bind Gram-positive and -negative bacteria, fungi, and protozoa (10,11). NETs form web-like structures that imprison microorganisms and prevent them from spreading. In addition, the chromatin released either by dying or activated living neutrophils, is covered with histones, granular enzymes such as elastase, and MPOs that kill microorganisms (12). NETs not only trap and kill microbes, but they also release LL37, an antimicrobial peptide, and the high-mobility group box protein 1 (HMGB1), which are important activators of plasmacytoid dendritic cells (DCs) via toll-like receptor 9 (TLR-9) (13). On the other hand, NETs can activate T cells by lowering their activation threshold in a TLR-9 independent way (14).

Another important property of neutrophils is the release of cytokines that can promote either pro- or anti-inflammatory activity depending on their combination and concentration. These cytokines include interleukin (IL)-1α, IL-1β, IL-1RA IL-17A, IL-17F, and many others. Moreover, neutrophils can exert immunomodulatory activities by secreting cytokines involved in T-cell fate, including interferon (IFN)-γ, IL-12, IL-23, transforming growth factor (TGF)-β1, IL-4, and many others (15). Overall, the data show that neutrophils have an enormous capacity to influence immunity.

**Figure 2:**
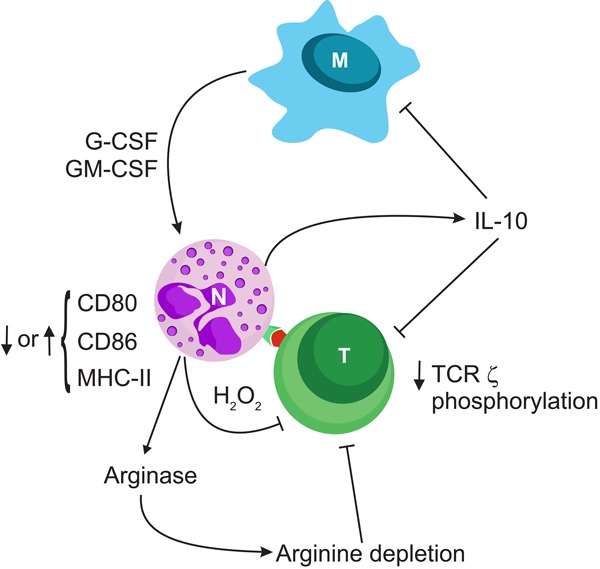
Neutrophils (N) cross-talk with other immune cells and are receptive of various innate or adaptive immunity system stimuli. This figure illustrates an example of the numerous possibilities for reciprocal regulation. Macrophages (M), along with many other cytokines, produce granulocyte colony-stimulating factor (G-CSF) and granulocyte-macrophage colony-stimulating factor (GM-CSF), which can in turn induce production of cytokines such as interleukin (IL)-10 by neutrophils. Under different conditions, neutrophils can modify the extent of major histocompatibility complex (MHC) and costimulatory molecule expression, produce reactive oxygen species and nitric oxide synthase, or consume arginine leading to inhibition of the T-cell (T) response. TCR: T-cell receptor.

Beyond their individual activity, neutrophils influence effector functions of other leukocytes in a direct or indirect manner through contact or cytokine production. Neutrophils and macrophages cross-talk through cytokines and chemokines, attracting each other to inflammatory sites. Also, neutrophils can transfer granule-derived molecules and ingested materials to macrophages. On the other hand, macrophages produce cytokines like granulocyte colony-stimulating factor (G-CSF) and granulocyte-macrophage colony-stimulating factor (GM-CSF), which prevent early neutrophil apoptosis. Apoptotic neutrophils and polymorphonuclear (PMN) leukocytes assist macrophages in the resolution of inflammation but can also activate their companion macrophages, if they are infected, contributing to tissue damage in longer lasting infections. Thus, through a combination of overlapping and complementary characteristics, the two most important professional phagocytes contribute to microbial clearance and resolution of inflammation or tissue damage (Figure 2) (16 17 18 19 19 20 21).

In addition to interacting with macrophages, neutrophils can also modulate DC functions. Interaction of lipopolysaccharide-stimulated polymorphonuclear (LPS-PMN) cells with DCs leads to upregulation of DC CD40, CD80, major histocompatibility complex (MHC) class II, and CD86 surface molecules, either by secreted soluble factors such as tumor necrosis factor (TNF)-α, or by cell-to-cell contact. LPS-PMNs also stimulate DC maturation through IL-12 and TNF-α production, which influence DC antigen presentation (22).

Another recently discovered neutrophil function involves interaction with, and modulation of, lymphocyte subsets. For example, neutrophils can act as accessory cells for natural killer (NK) cell activation in response to pathogens, and NK cells can modulate the survival, recruitment, and functional responses of neutrophils. It has been reported that NK-cell functions are impaired during neutropenia, confirming the importance of this cross-talk for human health (23).

Reciprocal interactions between neutrophils and T cells may promote mutual activation and upregulation of surface molecules such as MHC-II molecules, CD80, and CD86 in neutrophils. The functional consequence of increased expression of MHC-II and costimulatory molecules is the presentation of antigens to T cells. It is also possible for T cells to delay the death of neutrophils through secretion of cytokines like GM-CSF, allowing time for neutrophil antigen presentation (24). On the other hand, these interactions may also prevent T-cell activation in at least two different ways. The first is through production of H_2_O_2_, which is membrane-permeable and acts on neighboring cells. In T cells, peroxides prevent activation by decreasing T-cell receptor (TCR) ξ chain phosphorylation (25), which is a mechanism requiring neutrophil expression of the integrin, macrophage antigen (MAC)-1 (CD11b), which favors cell-to-cell interaction (26). The second mechanism by which neutrophils can inhibit T-cell activation is by the production of arginase (ARG) 1 and nitric oxide synthase (NOS) 2. Both enzymes impair T-cell activation by extracellular arginine depletion (27,28). Thus, in addition to modulating T cells through antigen presentation and cytokine production, neutrophils might impair T-cell activation by diminishing arginine availability and ζ-chain phosphorylation.

Human neutrophils are also a significant source of B-cell activating factor (BAFF) and APRIL (a proliferation-inducing ligand), which are cytokines that influence the survival, maturation, and differentiation of B cells (7). It was recently shown that a subpopulation of neutrophils, represented by a single phenotype, i.e., B-cell helper neutrophils, which produces BAFF, APRIL, CD40L, and IL-21, activates marginal-zone B cells, and promotes immunoglobulin class-switching, somatic hypermutation, and antibody production (29). The available data thus indicate a role for neutrophils in autoimmune and neoplastic B cell-dependent disorders (7).

It is reasonable to assume that there is a “silver lining” despite all the “evil” activities known to be practiced by neutrophils. Specific stimuli, the influence of various microenvironments, and interaction with other cell types all function to generate neutrophils with protective features.

Ways in which neutrophils influence the inflammatory environment in infection, tumors, or autoimmune responses, and regulate both innate and adaptive immune responses are reviewed below. We believe we will create a new face for this unfairly prejudged cell that has been traditionally considered to be only a suicide phagocyte.

## Neutrophils at sites of inflammation

### Neutrophils in infection

Neutrophils are known to control fungal and bacterial infections. Phagocytosis, release of NETs, and production of ROS and antimicrobial peptides are key activites in controlling and clearing *Staphylococcus* spp., *Streptococcus* spp., *Escherichia coli*, and *Mycobacterium tuberculosis* infections, for example. *Staphylococcus aureus* is a Gram-positive human commensal bacterium that, under certain conditions, can cause serious infections. The major outer surface component of Gram-positive bacteria is peptidoglycan, which, in addition to involvement in opsonization by immunoglobulin and complement, can induce oxidative bursts in neutrophils (26,30). To effectively kill *S. aureus* captured by phagocytosis, neutrophils release their granule contents (MPO, lactoferrin, lysozyme, defensins, cathelicidins, cathepsins, elastases, and proteases), which have antimicrobial or bacteriostatic properties that act along with ROS to kill bacteria (31). ROS production is an important, but not a unique, stimulus for the release of NETs (32). Despite all these defenses, some bacteria, such as *Streptococcus pyogenes*, employ strategies to escape from innate immune mechanisms.

Streptolysin O (SLO) from *S. pyogenes* inhibits bacterial transport into lysosomes, contributing to their escape (33). Also, some bacteria escape from or minimize the effectiveness of NETs through the expression of nucleases that degrade NET DNA (34).

*M. tuberculosis* is the main cause of tuberculosis, and neutrophils are the most numerous participants in the early response to lesions in mice (35) and the most abundant infected cells in the airways of infected humans (36). Previous studies have shown that the mycobactericidal capacity of neutrophils is independent of oxidative bursts, since ROS inhibitors did not affect bacterial killing. However, neutrophils exposed to *M. tuberculosis* release NETs that cannot kill the extracellular mycobacteria (37), but do restrict their dissemination and activate adjacent macrophages (18). Some studies suggest that neutrophils do not play an important role in the early phase of the disease, causing more tissue injury than protection from infection. However, neutrophils can help macrophages to kill mycobacterium by transferring live bacteria, cathelecidin, and lipocalin to macrophages (19).

Neutrophils also have a crucial role controlling fungal infections such as candidiasis. The genus *Candida* includes a few fungal species that can colonize the vaginal cavity, the gut, or the oral mucosa, even in healthy individuals (38). However, in people with certain infections such as human immunodeficiency virus (HIV), or those receiving antibiotics or immunosuppressive drugs, *Candida* spp. can be pathogenic and injure the host (39). When overgrown, *Candida* spp. activate neutrophils through a mannose-binding lectin pathway (40), toll-like receptors (TLR), or Fcγ receptors (mainly FcγRIIa) to undergo degranulation, phagocytosis, and NADPH oxidase assembly (41). NETs are crucial for toxic activity against *Candida* after phagocytosis because they contain calprotectin, which sequesters Mn^2+^ and Zn^2+^, which are essential for fungal metabolism (42). When phagocytized, *Candida albicans* can elevate the pH, which induces yeast-to-hyphae transition and leads to piercing, leakage, and escape from macrophages. However, neutrophils can inhibit *Candida* filamentation (20). Although the importance of bacterial and fungal killing by neutrophils is beyond question, it should be noted that all these features could turn against the host and worsen inflammation or autoimmune diseases like rheumatoid arthritis (RA) or systemic lupus erythematosus (SLE).

### Neutrophils in autoimmune diseases

**Figure 3:**
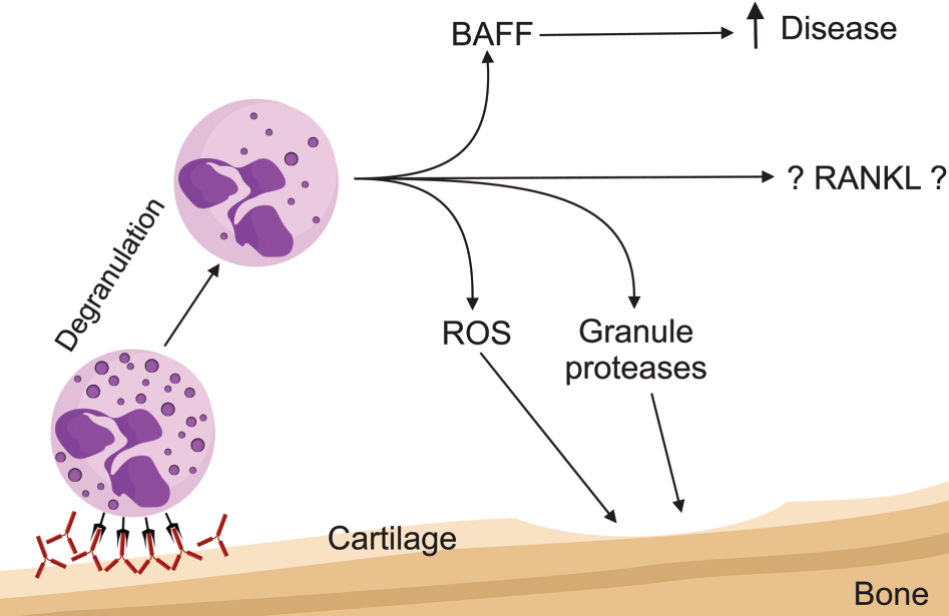
Neutrophils in autoimmune disease with rheumatoid arthritis as an example. Immunocomplexes activate neutrophils, which in turn release proteases damaging the cartilage. Induction of oxidative bursts generates reactive oxygen species (ROS), which also directly damage the articular cartilage. In addition, B-cell activating factor (BAFF) secretion activates B cells. Receptor activator of nuclear factor kappa-B ligand (RANKL) production by neutrophils has been shown in mature peripheral neutrophils. In bone, preliminary data indicate that RANKL derived from neutrophils in the bone marrow might participate in bone metabolism.

RA is a systemic inflammatory disorder that primarily affects the joints, causing pain and loss of function. Neutrophils from the blood of RA patients are primed to secrete high levels of ROS and cytokines (43). Activation of neutrophils through recognition of immune complexes by FcγRs induces degranulation with an increase in granule proteins in the synovia, leading to cartilage damage. ROS production is also augmented, which increases NET release and exposes granule contents and cytoplasmic and citrullinated autoantigens in the joints (44). These neutrophils can also secrete high levels of receptor activator of nuclear factor kappa-B ligand (RANKL) (45) and BAFF (46), which activate osteoclasts and B cells, respectively (Figure 3). In addition, neutrophils from RA patients upregulate MHC-II expression and increase the antigen presentation ability of neutrophils, leading to T-cell activation (47). The contribution of neutrophils to RA pathology can be seen in Felty’s syndrome (a severe form of RA) where the diagnostic findings include splenomegaly, high neutrophil counts, and autoantibodies against PAD-4, an arginine deaminase that converts arginine to citrulline that bind to neutrophils and NETs (48).

SLE is an autoimmune disease caused by type II and type III hypersensitivity due to deposition of immune complexes. Although lymphocytes are the cell type most closely involved in disease pathogenesis, neutrophils can modulate disease severity. Neutrophils from SLE patients present reduced phagocytic function (49) and increased release of NETs, antimicrobial peptides, double-stranded DNA (dsDNA), and a series of danger-associated molecular patterns (DAMPs) even in the absence of infection (50). SLE patient sera increase neutrophil aggregation (51), modulate oxidative burst, and are rich in neutrophil bactericidal proteins and autoantibodies against dsDNA that coat NETs and block its degradation by DNAses. NETs also induce DCs to produce IFN-α, IL-18, and IL-1β, which are responsible for positive feedback loops favoring NET release and inflammation associated with worsening of SLE (52).

In experimental autoimmune encephalomyelitis (EAE), an animal model of multiple sclerosis (MS), neutrophils are the first inflammatory cells to appear in the central nervous system. Upon stimulation by IL-1β, they infiltrate the spinal cord (53) and disrupt the blood-spinal cord barrier through ROS production that disrupts tight junction components (54). Following disruption of the blood-spinal cord barrier, Th17 lymphocytes infiltrate the CNS and synthesize chemokine (C-X-C motif) ligand 1 (CXCL) 1 and CXCL2 and TNF-α, GM-CSF, and IFN-γ, which can activate neutrophils to produce cytokines and ROS, upregulate MHC-II, and degranulate (55).

It seems that the effects of granulocyte bactericidal activity in the worsening of autoimmune diseases are not model- or disease-specific but general features of inflammatory syndromes.

## Tumor-associated neutrophils

The importance of neutrophils in the establishment, development, and spread of cancers is increasingly appreciated. The neutrophil/lymphocyte ratio is used as a prognostic factor in colorectal (56) and non-small-cell lung cancers (57). Infiltration of neutrophils is seen in more aggressive types of tumors such as pancreatic adenocarcinomas (58), but a high neutrophil count is associated with a favorable prognosis in gastric cancer (59). Tumor-associated neutrophils (TANs) have been observed in both animal models and humans, where they accumulate in blood vessels associated with primary tumors and metastatic sites. However, their role in tumor progression or eradication, and in metastasis establishment is still controversial (60,61).

Contradictory evidence can be partly explained by the high plasticity of neutrophils in response to primary tumors. Neutrophils make up a significant portion of the infiltrating inflammatory cells in different cancer models. They move into tumors under the influence of chemokines, cytokines, and cell adhesion molecules produced within the tumor microenvironment. They specialize under the direct influence of factors secreted by tumor cells, acquiring various phenotypes and functions (62). TANs in turn can then influence the tumor niche through the release of cytokines (e.g., TNF-α, IL-1β, IL-12), chemokines (members of the CXC and CC subfamilies), ROS, and growth factors. They alter the composition of the tumor microenvironment by stimulating angiogenesis, participating in polarization phenotypes, and recruiting other inflammatory cells (7,63,64). A description of TAN subtypes N1 and N2 illustrates how the tumor microenvironment can influence the phenotype of these cells. In the presence of TGF-β, resident TANs may acquire a protumor N2 phenotype. However, if TGF-β is blocked by administration of monoclonal antibodies, the neutrophils acquire an anti-tumor N1 phenotype. N1 neutrophils can be identified by hypersegmented nuclei, increased expression of intercellular adhesion molecule (ICAM) and TNF-α, and ability to activate CD8^+^ T lymphocytes. N2 neutrophils are characterized by high expression of arginase, CCL2 and CCL5 chemokines, and ability to inhibit effector T-cell functions (60).

G-CSF released from tumors can stimulate, expand, and mobilize Ly6C^+^Ly6G^+^ granulocytes that migrate to premetastatic sites, creating a protumorigenic microenvironment. These protumorigenic neutrophils support extravasation, survival, growth, and establishment of metastatic tumor cells. Pretreatment of animals with rG-CSF (recombinant murine protein) is sufficient to mimic the neutrophil expansion and the pre-metastatic microenvironment initiated by the primary tumor, reinforcing the proposed role of tumor-produced G-CSF (65). In a mouse model of mammary adenocarcinoma, myeloid-granulocytic CD11b^+^Gr1^+^ cells significantly increased in the lungs prior to tumor arrival (66). When in the lungs, neutrophils decreased IFN-γ production and produced large amounts of MMP9 and cytokines, which together created an environment permissive to the establishment of metastasis due to reduced immune protection and neovascularization. In that model, depletion of neutrophils significantly improved the immune response of the host and inhibited lung metastasis (66). Moreover, it has been demonstrated that cancer cells can stimulate neutrophils to produce oncostatin-M, which in turn increases secretion of vascular endothelial growth factor (VEGF) by tumors, promoting angiogenesis and neovascularization (67), adding to the cross-talk between tumor cells and neutrophils.

In contrast to the above findings, other investigators have described neutrophil antitumor activity associated with inhibition of the establishment of metastatic foci. In a renal carcinoma model (68), neutrophils were attracted to the lung (the most common site of metastasis in that model) by chemokines secreted by tumor cells. Those neutrophils were highly cytotoxic, creating an immunological, antimetastatic barrier preventing the establishment and growth of metastatic cells (68). It has already been suggested that neutrophils stimulated by murine breast cancer accumulate in the lung during the premetastatic stage (69). Those neutrophils acquire a cytotoxic phenotype and, by secreting H_2_O_2_, promote the generation of a protective shield through the elimination of metastatic cells. In this context, CCL2 secreted by tumors are critical mediators of neutrophil attraction. Neutrophil depletion of tumor-bearing animals increases the activation status of CD8^+^ T cells, supporting the idea that N2 TAN can function in an immune-suppressive fashion (60). Myeloid-derived suppressor cells (MDSC) are another TAN phenotype, which comprise a heterogeneous group of cells of myeloid origin, including neutrophils. MDSC are characterized mainly by their immature state in the periphery and for the ability to suppress T-cell responses (63). Increased MDSC in peripheral blood correlates with increased tumor burden and poor prognosis in cancer patients (70,71). MDSC are generated in bone marrow in response to factors secreted by tumor cells themselves, such as G-CSF, GM-CSF, IL-6, IL-1β, prostaglandin (PG) E2, VEGF, and TNF-α, and are recruited to the site of the primary tumor and secondary lymphoid organs (lymph nodes, spleen) by chemokines such as CCL2, CXCL12, and CXCL5. Through their suppressive mechanism, MDSC facilitate tumor establishment and propagation. These cells produce ARG1, NOS2, indoleamine 2,3-dioxygenase (IDO), and various immunosuppressive cytokines, which together inhibit cytotoxic T lymphocytes, NK, and dendritic cells and favor the expansion of regulatory T cells in the primary tumor niche. Granulocytic-myeloid derived suppressor cells express large amounts of ROS and small amounts of nitric oxide (NO), unlike the phenotype of monocytic myeloid suppressor cells, which express mainly NO and small amounts of ROS (63,72). Despite their phenotypic differences, both cell types suppress adaptive antitumor responses in several murine cancer models.

In summary, the role of tumor-associated neutrophils is ambiguous. Neutrophils are included in the inflammatory infiltrates in several types of cancer, and, depending on the stimuli to which they are exposed, they acquire either pro- or antitumorigenic roles. A better understanding of the mechanisms by which these cells act to promote or inhibit primary and secondary tumor growth is needed in order to develop successful therapeutic strategies based on stimulation of antitumor immune responses.

## Neutrophils as immunosuppressive cells

As noted above, only recently has it been accepted that neutrophils can have multiple phenotypes, including the classification as suppressor neutrophils. Little is known about the occurrence and induction of these differing cellular functions. We now know that in addition to their antimicrobial functions, neutrophils are able to drive and modulate the adaptive immune system. One possible reason for the delay in finding these multiple subtypes was the belief that neutrophils were short-lived suicidal cells that performed their antimicrobial duties and subsequently underwent apoptosis - and that was all! Evidence of increased survival, or lifespan, stimulated the search for additional phenotypes or subtypes.

Although the gold standard for identification of neutrophils is light microscopy, a number of markers can be used to detect different phenotypes. There is no consensus on the suppressor subtype, for which the most precise identification is functional (2). Markers like CD62L low and CD11b high are associated with suppressor phenotypes, and others such as CD11c, CD32, CD35, CD45, and CD66b can be upregulated in suppressor neutrophils. However, there is no known unique or specific suppressor phenotype profile (27).

As previously mentioned, there are published data describing neutrophils as suppressor cells. Some mechanisms have been identified and their relevance has been described, such as downregulation of the TCR ζ chain due to the consumption of L-arginine, which is important for T-cell proliferation (25). Regarding infections, three different types of neutrophils with differing susceptibility to infection with *Staphylococcus aureus* have been described in mice (21). In addition to normal polymorphonuclear neutrophils (PMN-N), there were at least two distinct subtypes (PMN-I and PMN-II). The suppressor subtype (PMN-II) could express TLR2/TLR4/TLR7/TLR9, and low levels of MPO, and could even promote the generation of M2 macrophages (21). Production of suppressor cytokines by neutrophils has been described, such as IL-10 in *Candida albicans* infections (73) and IL-22 associated with inhibition of colitis (74). Other investigators have reported that PMNs may present themselves as low-density granulocytes (LDGs) acting as T-cell suppressors in antitumor responses (34), during pregnancy (75), in seropositive HIV patients (76), in asthma (77), in RA and SLE patients (78), and in graft versus host disease (GVHD) (79). Under such conditions, production of H_2_O_2_ by neutrophils downregulates the TCR ζ chain, thereby inhibiting the synthesis of cytokines by T lymphocytes. Our group has characterized a suppressor LDG that is generated after treatment with G-CSF and inhibits GVHD (80). These cells produce relatively high concentrations of H_2_O_2_, express high levels of nitric acid synthase (NOS) 2 mRNA, are degranulated, and have low levels of MPO. Other molecules important for T-cell activation (e.g., CD80, CD86, and MHC-II) are also expressed at low levels in these LDGs (Perobelli SM, Mercadante AC, Galvani RG, Gonçalves-Silva T, Alves APG, Pereira-Neves A, Benchimol M, Nóbrega A, and Bonomo A, unpublished data) (Figure 2).

The ability of neutrophils to suppress T-cell activation is reinforced by regulatory T cells (Tregs), which are less sensitive than conventional T cells to H_2_O_2_ suppression (81). There is also evidence that Tregs can be induced by LDGs (Perobelli SM, Mercadante AC, Galvani RG, Gonçalves-Silva T, Alves APG, Pereira-Neves A, Benchimol M, Nóbrega A, and Bonomo A, unpublished data).

Production of cytokines is the most accurate indicator of cell specialization. It is known that murine neutrophils can produce several cytokines and chemokines such as IL-12, IL-10, IL-4, TNF-α, IL-1β, IL-22, CXCL1, CCL-2, and CCL-3 (74,82). Neutrophil subtypes that produce IL-10 have been described (21,73,). It is known that IL-10 is a potent anti-inflammatory cytokine produced by a variety of cells, including B cells, mast cells, eosinophils, macrophages, DCs, and a large number of T-cell subtypes in addition to neutrophils. This cytokine negatively regulates the synthesis of proinflammatory chemokines and cytokines such as IL-1, IL-6, and TNF-α. Furthermore, it may also regulate the synthesis of nitric oxide, collagenase, and gelatinase (87). We have shown that G-CSF treatment generates splenic neutrophils that inhibit GVHD, but if neutrophils were obtained from IL-10 knockout mice, this protection was largely abolished, showing the importance of donor-generated neutrophil-derived IL-10 to inhibit GVHD. The mechanism of action is not yet known, and does not exclude the possibility of joint action with another cytokine (Perobelli SM, Mercadante AC, Galvani RG, Gonçalves-Silva T, Alves APG, Pereira-Neves A, Benchimol M, Nóbrega A, and Bonomo A, unpublished data).

However, the published literature on the production of IL-10 by human neutrophils is conflicting. Despite the fact that several studies (21,73,83,84) have shown that mouse neutrophils produce IL-10 in response to a variety of infections, and the data are widely accepted, only two groups of investigators were able to reproduce those results using human neutrophils (85,86).

The reported differences in IL-10 production by mouse and human neutrophils may result from regulatory processes. Many cell types express IL-10 mRNA, but not all make detectable amounts of protein, and the extent of protein expression is variable. Much of the variation can be explained by post-transcriptional mechanisms. Protein production is related to stability of the 3'-UTR region of IL-10 mRNA, which is usually unstable, but depending on the stimulus (e.g., the tumor promoter PMA) the mRNA is stabilized, leading to protein production (88).

Another important neutrophil-mediated suppression mechanism is secretion of IL-22. This cytokine contributes to the maintenance of intestinal epithelium integrity, protecting against barrier breakdown (74). IL-22 is a member of the IL-10 cytokine family. It is produced by many different cells types, including Th17, Th22, NK, Tγδ, ILC (innate-like lymphocyte cells), and neutrophils (89). IL-22 plays a critical role in local modulation of inflammation in certain organs, and contributes to the integrity of the intestinal mucosa, and generation of a protective response against extracellular pathogenic bacteria (90). In a mouse model of colitis, Zindl et al. (74) described the role of IL-22-producing neutrophils in intestinal protection. Transfer of IL-22-competent neutrophils to IL-22-deficient animals protected them from dextran-induced colitis and induced the production of antimicrobial peptides, including the mucosal lectin RegIIIβ and the calcium-binding protein S100A8.

## Concluding remarks

In addition to the prevailing view of neutrophils as effectors of the immune response, we have reviewed some recently described roles. Evidence of cross-talk with other cell types and modulation of T and B cells shows that there are undeniably different subpopulations of neutrophils, or at least different stages of development and/or activation. Identifying neutrophil phenotypes and subtypes is of the utmost importance within the context of applied immunology, and segregation of these subtypes allows for application in clinical practice.

**Figure 4:**
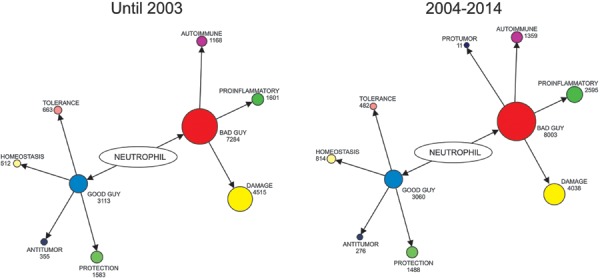
Representative image illustrating the view of neutrophils as “bad guys”. Searches of the Medline database (PubMed) using a broad strategy were preformed to identify studies related to “good” and “bad” neutrophil profiles. The number of publications returned in each search was entered into an igraph¯ library creating the representation depicted. The relationships (or edges) between the terms and neutrophils are presented by arrows and the terms are presented by circles (or nodes). The size of each is proportional to the number of publications returned. On the left side, the graph shows the publications until 2003 and on the right, from 2004 until 2014. Data were collected on November 15, 2014.

During the last 10 years (2004-2014), 8003 articles have been published interpreting neutrophils as “bad” guys contributing to disease pathology; while only 3060 described neutrophils as “good” guys, or contributing to disease protection or resolution (Figure 4). A search for articles published up to the year 2003 revealed 7284 papers pointing to the “bad” and 3113 to the “good”, which is not very different from what has been published in the last 10 years (Figure 4). The results of a search on the keywords “neutrophils AND protumorigenic” suggested that many tumor-helping neutrophil interactions have only recently been identified (Figure 4). Of note, and related to the topics addressed in this present review, almost twice as many papers on proinflammatory neutrophils available in the PubMed database have been published in the last 10 years than all the preceding years. When “homeostasis AND neutrophils” were searched, 60% more papers were published in the past 10 years than previously. These suggest a better appreciation of the regulatory role of neutrophils has developed in recent years, regardless of the potential pathological significance. The majority of the “good” activities of neutrophils relate to papers dealing with protection, which makes sense because their best-known effector function is pathogen elimination. Recognition of neutrophils as suppressors of inflammation to maintain homeostasis, tolerance, or even anti-tumorigenic activity has not increased as rapidly as the other aspects reviewed (Figure 4). We believe that in the near future, studies of suppressor neutrophils and their particular features will be of benefit to patients with immune-related diseases.

Overall, this review points to neutrophils as cells with phenotypic plasticity that can influence the microenvironments that they migrate to. This picture is in line with a cell type that has long been known for its importance for acute protection of our body, but was not appreciated as playing an important role in immune regulation, particularly immune suppression.
